# Understanding the Human RECQ5 Helicase—Connecting the Dots from DNA to Clinics

**DOI:** 10.3390/cells12162037

**Published:** 2023-08-10

**Authors:** Chiefe Mo, Yukari Shiozaki, Kenneth Omabe, Yilun Liu

**Affiliations:** Department of Cancer Genetics and Epigenetics, Beckman Research Institute of City of Hope, 1500 East Duarte Road, Duarte, CA 91010-3000, USA

**Keywords:** RECQ5, transcription, replication, DNA repair, genomic instability

## Abstract

RECQ5, a member of the conserved RECQ helicase family, is the sole human RECQ homolog that has not been linked to a hereditary developmental syndrome. Nonetheless, dysregulation of RECQ5 has emerged as a significant clinical concern, being linked to cancer predisposition, cardiovascular disease, and inflammation. In cells, RECQ5 assumes a crucial role in the regulation of DNA repair pathways, particularly in the repair of DNA double-strand breaks and inter-strand DNA crosslinks. Moreover, RECQ5 exhibits a capacity to modulate gene expression by interacting with transcription machineries and their co-regulatory proteins, thus safeguarding against transcription-induced DNA damage. This review aims to provide an overview of the multifaceted functions of RECQ5 and its implications in maintaining genomic stability. We will discuss the potential effects of clinical variants of RECQ5 on its cellular functions and their underlying mechanisms in the pathogenesis of cancer and cardiovascular disease. We will review the impact of RECQ5 variants in the field of pharmacogenomics, specifically their influence on drug responses, which may pave the way for novel therapeutic interventions targeting RECQ5 in human diseases.

## 1. The History of the RECQ-Associated Clinical Diseases

The RECQ family of DNA helicases is present in both prokaryotes and eukaryotes [1]. In humans, five RECQ homologs have been identified: *RECQL/RECQ1*, *BLM/RECQ2*, *WRN/RECQ3*, *RECQ4*, and *RECQ5*. Although there are multiple RECQ helicases in human cells, it is important to note that their sequence homologies are limited to the helicase domain [1]. This limited sequence similarity explains why mutations in different RECQ genes are associated with distinct diseases and why the clinical manifestation of those mutations in a particular RECQ helicase cannot be compensated for by the presence of other RECQ homologs.

The clinical significance of the RECQ family of DNA helicases was recognized long before the identification of the five RECQ genes in humans. In 1868, Dr. August Rothmund described the first RECQ-associated disease called Rothmund–Thomson syndrome (RTS), characterized by developmental abnormalities, premature aging, and cancer [1,2]. However, it took another 120 years to establish the connection between RTS and mutations in the *RECQ4* gene [3]. In 1904, Dr. Otto Werner reported a progeria syndrome in his doctoral dissertation, which would later be associated with WRN/RECQ3 and named Werner’s syndrome [4]. In 1954, another clinical case of congenital telangiectatic erythema with short stature was presented by Dr. David Bloom in New York, leading to the identification of Bloom’s syndrome [5,6]. The underlying cause of Bloom’s syndrome was revealed in 1965 when Dr. Bloom, in collaboration with Dr. James German, observed highly unstable chromosomes with increased DNA breakages, sister chromatin exchanges, and a quadriradial configuration in patients with Bloom’s syndrome [7]. The *BLM* gene, responsible for Bloom’s syndrome, was cloned in 1995 and found to share sequence similarities with the RECQ family of DNA helicases, including *RECQL/RECQ1* [8,9,10]. The third RECQ gene, initially known as *RECQ3*, was isolated during the search for gene mutations underlying Werner’s syndrome and later renamed *WRN* [11].

In 1998, the *RECQ4* and *RECQ5* genes, also known as *RECQL4* and *RECQL5*, respectively, were successfully isolated through a sequence homology search targeting the known RECQ family helicases [12]. Building upon the associations of BLM/RECQ2 and WRN/RECQ3 with Bloom’s syndrome and Werner’s syndrome, investigations were initiated to uncover hereditary diseases linked to RECQ1, RECQ4, and RECQ5. In 1999, Dr. Yasuhiro Furuichi and his team at the AGENE Research Institute in Japan made a significant breakthrough by establishing the connection between mutations in the *RECQ4* gene and a specific subset of RTS [3]. A few years later, RECQ4 mutations were also found to be associated with two additional developmental disorders, namely RAPADILINO syndrome and Baller–Gerold syndrome [13,14]. Another clinical milestone was recently reached, when mutations in the *RECQ1* gene were identified as the cause of the RECQL ONE (RECON) syndrome, characterized by a short stature, premature facial aging, xeroderma, and disproportional finger lengths [15]. Together, these findings have significantly contributed to our understanding of the clinical spectrum and genetic bases of RECQ helicase-related disorders, shedding light on the intricate roles played by these genes in human health and development.

RECQ5, the newest member of the human RECQ helicase family, does not have a known association with a specific clinical syndrome. Nevertheless, accumulating evidence suggests that RECQ5 plays distinctive roles in safeguarding genome integrity and preventing various diseases, including cancer, cardiovascular disorders, and inflammation. In this review, we summarize the current understanding of the clinical implications of RECQ5 polymorphisms or mutations. By correlating these genetic variations with the biochemical properties and cellular functions of RECQ5, we shed light on the potential mechanisms underlying RECQ5-related pathologies and pave the way for further investigations into RECQ5-associated therapeutic implications and potential targeted interventions.

## 2. *RECQ5* Gene Structure and Protein Biochemical Activities

**Figure 1:**
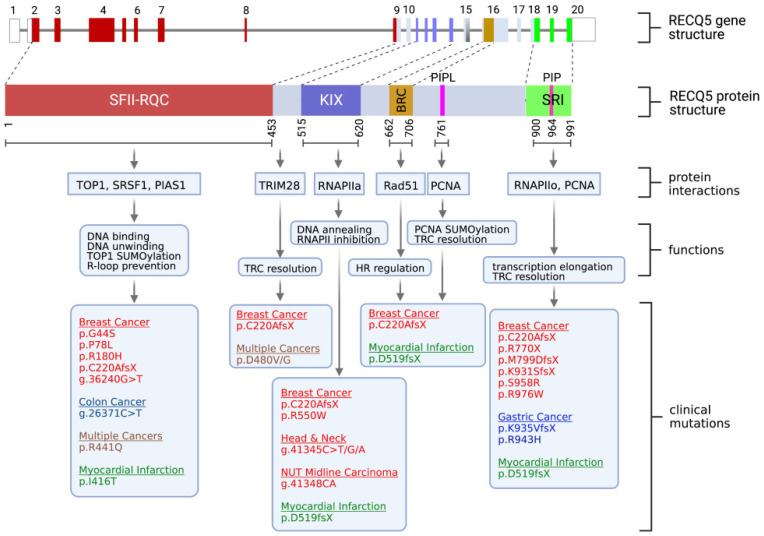
Summary of human *RECQ5* gene structure, RECQ5 protein domains, biochemical properties, cellular functions, and *RECQ5* clinical variants. (Top) Gene structure of the human *RECQ5* gene, including exons and introns, and the RECQ5 protein domains, including the Superfamily helicase II (SFII), RECQ-C-terminus (RQC), KIX, BRC, PIP-L, PIP and SRI domains. (Middle) Summary of the biochemical properties and functions of the RECQ5 domains. (Bottom) RECQ5 variants that have been linked to cancer or cardiovascular disease.

The human RECQ5 gene consists of 20 exons, 19 of which are coding exons spanning approximately 40 kb on chromosome 17 (Figure 1, top). The alternative splicing of RECQ5 mRNA results in three observed isoforms: RECQ5α, RECQ5β, and RECQ5γ [16]. Northern blot analysis indicates that RECQ5β mRNA is the predominant isoform in human cells, and its protein product localizes specifically to the nucleus [12,16]. The RECQ5 protein product encoded by the RECQ5β mRNA isoform is a 991 amino acid (aa) polypeptide, featuring a conserved helicase domain at the amino (N)-terminal region of the protein (Figure 1). Like other RECQ helicases, the catalytic core of RECQ5 helicase activity contains superfamily 2 (SFII) helicase and RecQ C-terminal (RQC) motifs, the latter being exclusive to the RECQ family [17,18,19].

The helicase domain of human RECQ5 demonstrates the ability to unwind various DNA structures, including partial duplex DNA, splayed arms, synthetic DNA fork structures, G-quadruplexes, and Holiday Junctions, in an ATP- and Mg^2+^-dependent 3′-5′ direction [18,20,21]. The binding and hydrolysis of the ATP co-factor induces a conformational change from a closed to an open state [22]. Apart from its DNA-unwinding activity, RECQ5 also exhibits intrinsic DNA strand-annealing activity, mapped to a region between residues 561 and 651 downstream of the SFII-RQC domain (Figure 1) [18,21]. This annealing reaction is inhibited by Replication Protein A (RPA), a single-stranded DNA (ssDNA)-binding protein that binds to the unwound ssDNA product, preventing its reannealing to the complementary strand [18]. Its inhibition in vitro may involve a direct protein–protein interaction between RECQ5 and RPA-coated ssDNA [23], as the inhibitory activity is specific to RPA and not to the *E. coli* ssDNA-binding protein SSB [18].

## 3. RECQ5 Functions in DNA Repair

The *RECQ5* gene has been identified in various multicellular organisms, including *Caenorhabditis elegans* (*C. elegans*) [24], Drosophila [25,26], mice [27], and chicken [28]. This allows for the study of RECQ5 deficiency in different model organisms, providing valuable insights even in the absence of direct links to human diseases. In *C. elegans*, although RECQ5 deficiency does not impact development, it does result in a shortened lifespan [29]. Because *C. elegans* RECQ5 is ubiquitously expressed and particularly enriched in the intestine compared to other organs, it is possible that in the absence of RECQ5, the accumulation of DNA damage in the intestine resulting from oxidative stress caused by environmental insults and food metabolism contributes to a shortened lifespan [29]. Similarly, in a mouse model with the deletion of the *Recq5* gene, no developmental abnormalities were observed, but these mice exhibited a strikingly high susceptibility to cancer at an older age compared to their wild-type counterparts [30]. At the cellular level, *Recq5* knockout (KO) mouse embryonic stem cells display an increase in sister chromatid exchanges (SCEs) due to an elevated frequency of homologous recombination (HR) in repairing DNA double-stranded breaks (DSBs) [30], resembling Dr. German’s initial observation in cells derived from Bloom’s syndrome patients [7]. Interestingly, in chicken DT40 cells, *RECQ5* deletion alone does not lead to significant increases in SCEs. However, when combined with a *BLM*^−/−^ mutation, *RECQ5-BLM* double-mutant cells exhibit significantly higher SCE levels compared to *BLM* single-mutant or *RECQ5* single-mutant cells. This suggests that RECQ5 functions as a backup for BLM in suppressing SCEs in chicken cells [28]. Nonetheless, several studies support the notion of a non-redundant role of RECQ5 in DNA damage repair, at least in mammalian cells [30,31,32].

RAD51 is essential for the strand invasion and pairing step of HR, and RECQ5 plays a crucial role in limiting SCEs by disrupting RAD51 presynaptic filaments during the initiation step of HR [30]. This activity is supported by the observation that RECQ5 localizes to DSBs [33,34], and its expression is inversely correlated with the number of RAD51 foci per cell [30]. The anti-RAD51 recombination function of RECQ5 is mediated through a direct interaction between RAD51 and the unique C-terminus of RECQ5 [35]. Sequence analysis has revealed that this RAD51-interacting domain of RECQ5, spanning residues 662 to 706, contains a BRC repeat motif like those found in breast cancer gene 2 (BRCA2), which interacts with RAD51 to promote HR in DSB repair [36]. Single-molecule studies have indicated that RECQ5 translocates along the RAD51 presynaptic filament in an ATP-dependent manner. This movement is believed to facilitate the disassembly of the filament and to limit the subsequent steps such as strand invasion and pairing to form a D-loop structure [23]. Additionally, by removing RAD51 from stalled replication forks, RECQ5 promotes access to the MUS81-EME1 flap endonuclease, which cleaves the stalled replication fork to resolve it [37]. Importantly, the dissociation of RAD51 filaments by RECQ5 also directs the repair of DSBs toward the synthesis-dependent strand annealing (SDSA) pathway that results in a noncrossover or alternative homology-dependent repair (HDR) utilizing a ssDNA donor independent of RAD51 [38,39].

## 4. RECQ5 Function in Transcription

In 1998, when the *RECQ5* gene was cloned, researchers initially expected it to be mainly involved in DNA repair based on the sequence homology to other RECQ homologs. It was, therefore, a surprise when RECQ5 was also linked to transcription regulation. Specifically, RECQ5 has been identified as an RNA polymerase II (RNAPII)-associated protein [40,41,42]. Through domain mapping, two regions located at the C-terminal portion of the RECQ5 protein were identified as being responsible for the direct interaction with RPB1, the largest subunit of RNAPII [40,43]. Sequence analysis revealed that these two RNAPII interaction domains share sequence homology with the KIX domain and SET2-Rpb1 interacting (SRI) domain found in transcription factors CBP and SET2, respectively (Figure 1) [43]. The KIX domain primarily interacts with RNAPIIa, the inactive and non-phosphorylated form of RNAPII, while the SRI domain is critical for the interaction with active, hyperphosphorylated RNAPIIo [41,43,44,45]. This distinct preference in interactions with different forms of RNAPII allows RECQ5 to function as both a negative and positive regulator of RNAPII-dependent transcription. The binding of RECQ5 via the KIX domain inhibits RNAPII at both the initiation and elongation steps in in vitro transcriptional assays, and this inhibition does not require RECQ5 ATPase activity [46]. Structural analysis suggests that the KIX domain binds to RNAPII in a manner similar to that of the TFIIS transcription factor, which is important for transcription elongation, thereby sterically blocking transcription elongation and preventing TFIIS interaction with RNAPII [47]. Given that the KIX domain overlaps with residues 561–651, which are responsible for single-stranded annealing activity [18,21], it would be worth investigating if RECQ5’s annealing activity contributes to transcriptional inhibition by preventing ssDNA formation at the promoter region, thereby reducing promoter accessibility to RNAPII.

In addition to its negative regulation of RNAPII-dependent transcription via the KIX domain, several studies support the role of RECQ5 during transcription elongation through the SRI domain [45]. RNAPII-dependent transcription is initiated by the TFIIH-mediated Ser5 phosphorylation of the RNAPII C-terminal repeat domain (CTD), followed by the Ser2 phosphorylation of the CTD as RNAPII transitions into the elongation mode [48]. The SRI domain of RECQ5 binds to hyperphosphorylated RNAPIIo proteins at both Ser2 and Ser5, and this binding correlates with the RECQ5 association with gene regions that are being actively transcribed [45]. One key function of RECQ5 in transcription elongation is to control the speed of RNAPIIo to prevent transcription stalling and backtracking [49].

## 5. RECQ5 Function in Transcription-Induced Replication Stress

Despite an increase in HR efficiency for repairing DSBs in *recq5* mutant cells, these cells exhibit an accumulation of spontaneous DSBs [30]. This suggests that RECQ5 also plays a crucial role in preventing the generation of DSBs in the first place. Subsequent investigations revealed that transcription is the major source of DSBs in the absence of RECQ5, and the RECQ5 SRI domain is critical in minimizing transcription-induced DSBs [44]. While transcription is essential for cell survival and growth, it poses a significant threat to DNA integrity. For example, during transcription, positively supercoiled DNA accumulates ahead of the RNAPIIo, while negatively supercoiled DNA is generated behind it. If left unresolved, these supercoils can impede the progress of RNAPIIo and hinder transcription. Additionally, the formation of RNA:DNA hybrids, known as R-loops, between the DNA template and newly synthesized RNA is driven by the negative supercoiling of DNA. Failure to properly resolve R-loops can result in the stalling of transcription and DNA replication forks, ultimately leading to the formation of DSBs [50]. Topoisomerase I (TOP1) is involved in preventing R-loops because TOP1 maintains proper DNA topology by removing supercoils during transcription [50,51]. However, TOP1 DNA cleavage activity itself can become harmful to cells, as it can lead to TOP1 being covalently trapped on the DNA due to DNA cleavage adjacent to misincorporated ribonucleotides or naturally aborted topoisomerase reactions, resulting in DNA nicks and trapped TOP1 molecules, which are major sources of mutagenesis [52,53,54]. RECQ5 plays a critical role in preventing R-loop-induced DSBs, and this is achieved through the induction of the SUMO1 conjugation of TOP1 [55]. SUMOylated TOP1 prevents R-loop formation by facilitating the binding of splicing factors to pre-messenger RNA (pre-mRNA) transcripts, preventing the invasion of RNA into the DNA template. Furthermore, by excising introns from pre-mRNA, the homology between newly synthesized mRNA and the DNA template is reduced, thereby decreasing R-loop stability [56]. The SUMOylation of TOP1, a process that is dependent on RECQ5, also suppresses TOP1-induced DSBs by inhibiting TOP1 catalytic activity [55]. In the event of replication fork stalling caused by an R-loop, RECQ5 also facilitates the disassembly of RAD51 filaments that may form at the stalled replication fork. This disassembly allows the replication fork to become accessible to MUS81-EME1 flap endonuclease cleavage, thereby alleviating supercoiled DNA buildup and destabilizing the R-loop at the collision site [37].

In addition to R-loops, the presence of chromatin-binding proteins, such as the RNAPII transcription machinery, can impede the progression of the replication fork, leading to fork collapse and the formation of DSBs [57,58]. DSBs induced by transcription–replication conflicts (TRCs) are a significant contributor to instability in common fragile sites (CFSs) [57,59,60]. CFSs are particularly prone to instability because they reside within gene regions that are predominantly transcribed during the S-phase of the cell cycle [59,61]. The detrimental impact of TRC-induced DSBs on human health is exemplified by the fact that CFS instability is associated with genomic rearrangements, a loss of heterozygosity, and microsatellite instability, all of which contribute to cancer pathogenesis [62,63]. Indeed, many genes located within CFSs are frequently deleted in cancer cells [64]. Importantly, genome-wide analyses have demonstrated a positive correlation between RECQ5 deficiency and genome rearrangements, or loss associated with CFS instability, underscoring the role of RECQ5 in TRC resolution [49]. Further mechanistic studies have shed light on how RECQ5 resolves TRCs by interacting with both RNAPIIo and proliferating cell nuclear antigens (PCNAs), an essential component of the DNA replisome [21,40,65,66,67]. The RECQ5 C-terminus contains PCNA-interacting proteins (PIPs) and PIP-like (PIP-L) motifs (Figure 1) [66,68]. Specifically, the interaction between PCNA and RECQ5 PIP-L recruits the TRIM28 SUMO E3 ligase, leading to the conjugation of SUMO2 to PCNA on actively transcribed chromatin [67]. This SUMO2-conjugated PCNA promotes the association of histone chaperones CAF1 and FACT with the replisome, facilitating histone exchange, enhancing repressive histone marks, and destabilizing the stalled RNAPIIo on chromatin [66]. The dissociation of RNAPIIo allows the stalled replication fork to restart, thereby avoiding catastrophic fork collapse and DSB formation. RECQ5 also facilitates the ubiquitin (Ub) conjugation of PCNA through the PIP motif within the SRI domain [68]. The helicase activity of RECQ5 may assist in unloading Ub-PCNA, enabling RNAPII to bypass the TRC site. It is important to note that RECQ5’s role in TRC resolution extends beyond gene regions transcribed by RNAPII, as the RECQ5 C-terminus also interacts with the largest subunit of RNAPI, preventing replication fork stalling at ribosomal DNA loci mediated by RNAPI [68].

## 6. RECQ5 Variants in Cancer

To date, no specific developmental syndrome has been directly attributed to mutations in the *RECQ5* gene. However, mutations affecting different domains of RECQ5 have been associated with an increased risk of cancer and cardiovascular disease. Studies involving *RecQ5* KO mice have revealed a high incidence of blood cancer and various solid tumors, indicating that RECQ5 functions as a broad-spectrum tumor suppressor [30]. To explore the connection between RECQ5 and cancer in humans, researchers have investigated *RECQ5* gene expression, polymorphisms, and mutations in relation to cancer risks. For instance, similar to the pattern observed in *C. elegans* [29], RECQ5 expression in humans is predominantly enriched in the gastrointestinal tract [69]. A key function of RECQ5 in the gastrointestinal tract is to suppress tumorigenesis, as low RECQ5 expression has been associated with colorectal cancer and gastric cancer, and this reduced expression is correlated with poor prognosis [70,71,72]. The link between reduced RECQ5 expression and the neoplastic transformation of gastrointestinal tract tissue has been further validated in APC (min/+) colorectal mouse models [73]. Reduced RECQ5 expression has also been observed in osteosarcoma, particularly in advanced tumor stages or low-grade tumors [74]. The precise mechanism by which RECQ5 regulates bone metastasis in this context remains to be elucidated.

It is worth noting that mutations in the tumor suppressor genes, *FHIT* and *WWOX*, which are located within FRA3B and FRA16D CFS, respectively, due to unresolved TRC-induced CFS instabilities have been observed at a high frequency in gastric and colon cancer [75,76]. These observations suggest that RECQ5 may prevent the pathogenesis and invasiveness of gastric cancer by suppressing CFS instabilities induced by TRCs. Indeed, whole-exome sequencing studies conducted on families with hereditary gastric cancer but lacking pathogenic mutations in the *CDH1* gene, which encodes E-Cadherin, have identified two RECQ5 variants, namely c.2828C>T and c.2806-2A>G (Figure 1, Table 1), in separate families [77]. Both *RECQ5* mutations are predicted to alter the protein-coding sequence within the SRI domain that is essential for TRC resolution [66,67]. In support of this connection, pathway analysis of gastric cancer has led to the identification of two genes encoding components of RNAPII that show a significant correlation with RECQ5 expression [71]. This finding further strengthens the link between the role of RECQ5 in transcription regulation and the development of gastric cancer.

Similarly, a number of deletion and missense mutations affecting the function of the RECQ5 SRI domain have been identified in breast cancer cases [78,79], suggesting the significance of the anti-tumor properties associated with the RECQ5 SRI domain across multiple cancer types. In a large case–control study conducted on a Spanish population, RECQ5 emerged as the only RECQ helicase gene with mutations associated with breast cancer risk [78,79]. In addition to the RECQ5 variants identified within the SRI domain, a significant number of missense mutations associated with breast cancer were found in the conserved residues within the SFII domain (Table 1) [78,79]. For instance, residue G44, located immediately upstream of the first of the seven SFII motifs, is conserved among all RECQ helicases except WRN/RECQ3 [12]. Similarly, residue P78 in the SFII Ia motif is shared by all RECQ helicases. Since the ATPase activity of RECQ5 plays a crucial role in anti-RAD51 recombinase activity [23], further biochemical analyses are necessary to determine the potential impact of the breast cancer-associated RECQ5 variants within the SFII and RQC domains on the ATPase activity of RECQ5 and its ability to disassemble the RAD51 filament on DNA. The functional relationship between RECQ5 and RAD51 in breast cancer is further supported by the studies showing that the overexpression of RECQ5, particularly when combined with low RAD51 expression, has been associated with increased invasion and migration in breast cancer [80]. This suggests that the excessive amount of RECQ5 may hinder RAD51 activity, leading to reduced HR efficiency and increased cancer aggressiveness. Interestingly, high expression of RECQ5 has also been linked to improved relapse-free survival in breast cancer patients [81]. These findings highlight the complex and context-dependent role of RECQ5 in breast cancer prognoses, indicating its potential as a prognostic marker that varies across specific breast cancer subtypes.

A number of single-nucleotide polymorphisms (SNPs) associated with RECQ5 have also been identified and correlated with susceptibility to various cancer types (Table 1). These SNPs, including rs820200, rs4789223, rs142406301, and rs74632503, fall within introns, and the impact of these SNPs on RECQ5 protein structure and expression at the protein level remains unclear. Nonetheless, rs4789223 has been linked to colon cancer and osteosarcoma [82,83], while rs820200 is associated with an increased incidence of breast cancer [84]. Notably, rs142406301 has a high recurrence in NUT midline carcinoma, an aggressive and fatal malignancy for which effective treatments have not been identified to date [85]. Interestingly, the rs74632503 variant shows a protective effect against head and neck cancer [86]. On the other hand, rs820196 involves a single nucleotide substitution within the RECQ5 coding sequence, resulting in a change from a negatively charged aspartic acid (D) side chain at the residue 480 to a non-polar glycine (G) or valine (V) residue. This D480G/V mutation has been associated with several cancer types, including breast, colon, laryngeal, and bone cancers [82,83,84,87,88,89,90,91]. This residue is situated between the SFII-RQC and KIX domains in a region where the interaction with the TRIM28 SUMO E3 ligase important for SUMO2-PCNA conjugation and TRC resolution was mapped [67]. Given that the secondary structure of this region is predicted to be disordered [92,93], the impact of the D480G/V mutation on the biochemical activity of RECQ5, the interaction with TRIM28, or potential post-translational modifications, such as nearby Y484 phosphorylation [94], is yet to be determined.

**Table 1:** Diseases associated with RECQ5 mutations and SNPs.

*Mutation/SNP*	*Effect*	*Domain(s) Affected*	*Syndrome*	*Cancer Type*	*Ref*
*rs4789223* g.26371C>T	intron variant			colon	[82]
*rs820200* g.36240G>T	intron variant			Breast	[84]
*rs74632503* g.41345C>T/G/A	intron variant			head & neck	[86]
*rs142406301* g.41348CA	intron variant			NUT midline carcinoma	[85]
c.130G>A	p.G44S	SFII-RQC		breast	[78,79]
c.233C>T	p.P78L	SFII-RQC		breast	[78]
c.539G>A	p.R180H	SFII-RQC		breast	[78]
c.657delC	p.C220AfsX	SFII-RQC, KIX, BRC, PIP-L, SRI, PIP		breast	[78,79]
c.1247T>C	p.I416T	SFII-RQC	myocardial infarction		[95]
	p.R441Q	SFII-RQC		multiple primary cancers	[96]
*rs820196* c.1439A>T; c.1439A>G	p.D480V/G		heart rate abnormality	breast, laryngeal, osteosarcoma, colon	[82,83,84,87,88,89,90,91]
c.1718insTG	p.D519fsX	KIX, BRC, PIP-L, SRI, PIP	myocardial infarction		[97]
c.1648C>T	p.R550W	KIX		breast	[78]
c.2308C>T	p.R770X	SRI, PIP		breast	[78,79]
c.2393dupC	p.M799DfsX	SRI, PIP		breast	[78,79]
c.2790C>T	p.K931SfsX	SRI, PIP		breast	[78,79]
c.2806-2A>G	p.K935VfsX	SRI, PIP		gastric	[77]
c.2828G>A	p.R943H	SRI		gastric	[77]
c.2874C>G	p.S958R	SRI		breast	[78,79]
c.2926C>T	p.R976W	SRI		breast	[78]

## 7. RECQ5 Variants in Heart Disease and Inflammation

In addition to its association with cancer, whole-exome sequencing studies have identified a specific mutation in the *RECQ5* gene that is highly prevalent in early myocardial infarction (MI) cases [97]. This mutation affects the acceptor splice site of intron 11, resulting in the skipping of exon 12 [97]. Consequently, the predicted protein product retains only the first 519 amino acids, lacking functional KIX, BRC, and SRI domains (Table 1; Figure 1). Furthermore, gastric cancer patients carrying the SNP rs820196, which leads to the RECQ5 D480V/G missense mutation and is associated with increased cancer risks, have been found to be more susceptible to heart rate changes during gastrectomy and peritoneal lavage [89]. Although the precise role of RECQ5 in heart disease has not been extensively studied, chromatin immunoprecipitation experiments have revealed RECQ5 localization to the gene body of the low-density lipoprotein receptor (LDLR) gene, and RECQ5 deficiency has been shown to alter LDLR expression [41]. LDLR plays a crucial role in regulating plasma cholesterol levels by removing circulating LDL through binding and internalization, and mutations in the LDLR gene have been linked to cardiovascular disease [98,99]. This association suggests that RECQ5 may play a role in maintaining proper cholesterol balance to prevent heart disease. Additionally, chronic inflammation contributes to atherosclerosis, a critical factor in the development and progression of cardiovascular disease [100]. Recent research indicates that RECQL5 may function to suppress lipopolysaccharide-induced inflammatory cytokine levels and inflammation-associated DNA damage [101]. Consequently, dysregulated inflammation due to altered RECQ5 function could potentially predispose individuals to heart disease.

## 8. Additional RECQ5 Variants in Humans

Numerous additional variants of the *RECQ5* gene have been identified in human populations, with some observed in individuals with congenital genetic disorders and others predicted to be pathogenic based on the change in the amino acid sequence [102,103]. Notably, no individuals among the more than 9000 sequences examined were found to be homozygous for the predicted pathogenic RECQ5 variants [103]. It is worth mentioning that Y723 is likely subjected to phosphorylation [94]. Nevertheless, the significance of these mutations in the context of genetic disorders or cancer has yet to be confirmed.

## 9. RECQ5 as a Therapeutic Target

Recent studies have shed light on the potential influence of RECQ5 variants in response to certain drugs. For instance, RECQ5 plays a role in promoting the SUMOylation of TOP1 during transcription, facilitating spliceosome recruitment to actively transcribed chromatin [55]. This modification also suppresses the catalytic activity of TOP1 in relaxing supercoiled DNA [55]. As the catalytic activity of TOP1 is crucial for the trapping of TOP1 by chemotherapeutic drugs such as topotecan and irinotecan, the deficiency of RECQ5 reduces TOP1 SUMOylation and increases TOP1 activity, rendering cells more sensitive to topotecan treatment [55,104]. Therefore, tumor cells with reduced RECQ5 expression, such as those in gastric cancer and osteosarcoma [71,72,74], may represent a cell population particularly susceptible to topotecan and irinotecan therapies.

Moreover, RECQ5 may be a therapeutic target for patients with myeloproliferative neoplasms (MPNs) harboring the V617F mutation in the Janus family of cytoplasmic non-receptor tyrosine kinase 2 (JAK2) [105]. The JAK2 V617F mutation induces replication stress, likely due to its oncogenic role in transcription induction, leading to an accumulation of TRC-associated DNA lesions. In this context, MPNs with the JAK2 V617F mutation depend on RECQ5 for the resolution of replication-associated DNA lesions for their survival [105]. Additionally, increased RECQ5 expression promotes tumor proliferation in urothelial bladder carcinomas [106]. Therefore, RECQ5 may be a potential therapeutic target for these cancers. To date, a small molecule derived from 1,3,4-oxadiazole has been discovered to inhibit RECQ5 helicase activity in vitro and sensitize RECQ5-positive cells, with an IC_20_ of 8.2 uM [107]. However, further validation is required to confirm the specificity of this small molecule to RECQ5 as opposed to other RECQ helicases, as computational analysis suggests its binding site is within the SFII domain [107], which is present in all known RECQ helicases. Small molecules targeting the protein–protein interaction domains in the C-terminal region of RECQ5, which are unique to RECQ5, may enhance selectivity toward RECQ5, providing an avenue for potential therapeutic development.

## 10. Conclusions

Over the past 25 years, a wealth of research has illuminated the complex functions of RECQ5 in DNA repair and transcriptional regulation, which are pivotal for maintaining chromosome integrity and controlling cell growth. Studies on RECQ5 variants may advance fundamental and clinical investigations on the impact of functional alterations in RECQ5 on human health and on effective therapeutic approaches tailored to individuals harboring specific variants. By leveraging this knowledge, we can pave the way for significant advancements in our understanding of the role of RECQ5 in various biological processes and optimize strategies to address the specific needs of individuals affected by these variants.

## Data Availability

No new data were created.

## References

[1] Liu Y. (2010). Rothmund-Thomson syndrome helicase, RECQ4: On the crossroad between DNA replication and repair. DNA Repair.

[2] Rothmund A. (1868). Ueber Cataracten in Verbindung mit einer eigenthümlichen Hautdegeneration. Arch. Ophthalmol..

[3] Kitao S., Shimamoto A., Goto M., Miller R.W., Smithson W.A., Lindor N.M., Furuichi Y. (1999). Mutations in RECQL4 cause a subset of cases of Rothmund-Thomson syndrome. Nat. Genet..

[4] (1985). On cataract in conjunction with scleroderma. Otto Werner, doctoral dissertation, 1904, Royal Ophthalmology Clinic, Royal Christian Albrecht University of Kiel. Adv. Exp. Med. Biol..

[5] Bloom D. (1954). Congenital telangiectatic erythema resembling lupus erythematosus in dwarfs; probably a syndrome entity. AMA Am. J. Dis. Child..

[6] Passarge E. (2016). James L. German, a pioneer in early human genetic research turned 90. Am. J. Med. Genet. A.

[7] German J., Archibald R., Bloom D. (1965). Chromosomal Breakage in a Rare and Probably Genetically Determined Syndrome of Man. Science.

[8] Ellis N.A., Groden J., Ye T.Z., Straughen J., Lennon D.J., Ciocci S., Proytcheva M., German J. (1995). The Bloom’s syndrome gene product is homologous to RecQ helicases. Cell.

[9] Puranam K.L., Blackshear P.J. (1994). Cloning and characterization of RECQL, a potential human homologue of the Escherichia coli DNA helicase RecQ. J. Biol. Chem..

[10] Seki M., Miyazawa H., Tada S., Yanagisawa J., Yamaoka T., Hoshino S., Ozawa K., Eki T., Nogami M., Okumura K. (1994). Molecular cloning of cDNA encoding human DNA helicase Q1 which has homology to Escherichia coli Rec Q helicase and localization of the gene at chromosome 12p12. Nucleic Acids Res..

[11] Yu C.E., Oshima J., Fu Y.H., Wijsman E.M., Hisama F., Alisch R., Matthews S., Nakura J., Miki T., Ouais S. (1996). Positional cloning of the Werner’s syndrome gene. Science.

[12] Kitao S., Ohsugi I., Ichikawa K., Goto M., Furuichi Y., Shimamoto A. (1998). Cloning of two new human helicase genes of the RecQ family: Biological significance of multiple species in higher eukaryotes. Genomics.

[13] Siitonen H.A., Kopra O., Kääriäinen H., Haravuori H., Winter R.M., Säämänen A.M., Peltonen L., Kestilä M. (2003). Molecular defect of RAPADILINO syndrome expands the phenotype spectrum of RECQL diseases. Hum. Mol. Genet..

[14] Van Maldergem L., Siitonen H.A., Jalkh N., Chouery E., De Roy M., Delague V., Muenke M., Jabs E.W., Cai J., Wang L.L. (2006). Revisiting the craniosynostosis-radial ray hypoplasia association: Baller-Gerold syndrome caused by mutations in the RECQL4 gene. J. Med. Genet..

[15] Datta A., Sommers J.A., Jhujh S.S., Harel T., Stewart G.S., Brosh R.M. (2023). Discovery of a new hereditary RECQ helicase disorder RECON syndrome positions the replication stress response and genome homeostasis as centrally important processes in aging and age-related disease. Ageing Res. Rev..

[16] Shimamoto A., Nishikawa K., Kitao S., Furuichi Y. (2000). Human RecQ5beta, a large isomer of RecQ5 DNA helicase, localizes in the nucleoplasm and interacts with topoisomerases 3alpha and 3beta. Nucleic Acids Res..

[17] Hamadeh Z., Lansdorp P. (2020). RECQL5 at the Intersection of Replication and Transcription. Front. Cell Dev. Biol..

[18] Garcia P.L., Liu Y., Jiricny J., West S.C., Janscak P. (2004). Human RECQ5beta, a protein with DNA helicase and strand-annealing activities in a single polypeptide. EMBO J..

[19] Andrs M., Hasanova Z., Oravetzova A., Dobrovolna J., Janscak P. (2020). RECQ5: A Mysterious Helicase at the Interface of DNA Replication and Transcription. Genes.

[20] Budhathoki J.B., Maleki P., Roy W.A., Janscak P., Yodh J.G., Balci H. (2016). A Comparative Study of G-Quadruplex Unfolding and DNA Reeling Activities of Human RECQ5 Helicase. Biophys. J..

[21] Kanagaraj R., Saydam N., Garcia P.L., Zheng L., Janscak P. (2006). Human RECQ5beta helicase promotes strand exchange on synthetic DNA structures resembling a stalled replication fork. Nucleic Acids Res..

[22] Newman J.A., Aitkenhead H., Savitsky P., Gileadi O. (2017). Insights into the RecQ helicase mechanism revealed by the structure of the helicase domain of human RECQL5. Nucleic Acids Res..

[23] Xue C., Molnarova L., Steinfeld J.B., Zhao W., Ma C., Spirek M., Kaniecki K., Kwon Y., Beláň O., Krejci K. (2021). Single-molecule visualization of human RECQ5 interactions with single-stranded DNA recombination intermediates. Nucleic Acids Res..

[24] Wilson R., Ainscough R., Anderson K., Baynes C., Berks M., Bonfield J., Burton J., Connell M., Copsey T., Cooper J. (1994). 2.2 Mb of contiguous nucleotide sequence from chromosome III of *C. elegans*. Nature.

[25] Sekelsky J.J., Brodsky M.H., Rubin G.M., Hawley R.S. (1999). Drosophila and human RecQ5 exist in different isoforms generated by alternative splicing. Nucleic Acids Res..

[26] Jeong S.M., Kawasaki K., Juni N., Shibata T. (2000). Identification of Drosophila melanogaster RECQE as a member of a new family of RecQ homologues that is preferentially expressed in early embryos. Mol. Gen. Genet..

[27] Ohhata T., Araki R., Fukumura R., Kuroiwa A., Matsuda Y., Abe M. (2001). Cloning, genomic structure and chromosomal localization of the gene encoding mouse DNA helicase RECQL5beta. Gene.

[28] Wang W., Seki M., Narita Y., Nakagawa T., Yoshimura A., Otsuki M., Kawabe Y.-I., Tada S., Yagi H., Ishii Y. (2003). Functional Relation among RecQ Family Helicases RecQL1, RecQL5, and BLM in Cell Growth and Sister Chromatid Exchange Formation. Mol. Cell. Biol..

[29] Jeong Y.S., Kang Y.L., Lim K.H., Lee M.H., Lee J., Koo H.-S. (2003). Deficiency of Caenorhabditis elegans RecQ5 homologue reduces life span and increases sensitivity to ionizing radiation. DNA Repair.

[30] Hu Y., Raynard S., Sehorn M.G., Lu X., Bussen W., Zheng L., Stark J.M., Barnes E.L., Chi P., Janscak P. (2007). RECQL5/Recql5 helicase regulates homologous recombination and suppresses tumor formation via disruption of Rad51 presynaptic filaments. Genes Dev..

[31] Hu Y., Lu X., Barnes E., Yan M., Lou H., Luo G. (2005). Recql5 and Blm RecQ DNA helicases have nonredundant roles in suppressing crossovers. Mol. Cell. Biol..

[32] Kim T.M., Son M.Y., Dodds S., Hu L., Luo G., Hasty P. (2015). RECQL5 and BLM exhibit divergent functions in cells defective for the Fanconi anemia pathway. Nucleic Acids Res..

[33] Popuri V., Huang J., Ramamoorthy M., Tadokoro T., Croteau D.L., Bohr V.A. (2017). RECQL5 plays co-operative and complementary roles with WRN syndrome helicase. Nucleic Acids Res..

[34] Ramamoorthy M., May A., Tadokoro T., Popuri V., Seidman M.M., Croteau D.L., Bohr V.A. (2013). The RecQ helicase RECQL5 participates in psoralen-induced interstrand cross-link repair. Carcinogenesis.

[35] Schwendener S., Raynard S., Paliwal S., Cheng A., Kanagaraj R., Shevelev I., Stark J.M., Sung P., Janscak P. (2010). Physical Interaction of RECQ5 Helicase with RAD51 Facilitates Its Anti-recombinase Activity. J. Biol. Chem..

[36] Islam M.N., Paquet N., Fox D., Dray E., Zheng X.F., Klein H., Sung P., Wang W. (2012). A variant of the breast cancer type 2 susceptibility protein (BRC) repeat is essential for the RECQL5 helicase to interact with RAD51 recombinase for genome stabilization. J. Biol. Chem..

[37] Di Marco S., Hasanova Z., Kanagaraj R., Chappidi N., Altmannova V., Menon S., Sedlackova H., Langhoff J., Surendranath K., Hühn D. (2017). RECQ5 Helicase Cooperates with MUS81 Endonuclease in Processing Stalled Replication Forks at Common Fragile Sites during Mitosis. Mol. Cell.

[38] Paliwal S., Kanagaraj R., Sturzenegger A., Burdova K., Janscak P. (2014). Human RECQ5 helicase promotes repair of DNA double-strand breaks by synthesis-dependent strand annealing. Nucleic Acids Res..

[39] Olson H.C., Davis L., Kiianitsa K., Khoo K.J., Liu Y., Knijnenburg T.A., Maizels N. (2018). Increased levels of RECQ5 shift DNA repair from canonical to alternative pathways. Nucleic Acids Res..

[40] Aygün O., Svejstrup J., Liu Y. (2008). A RECQ5–RNA polymerase II association identified by targeted proteomic analysis of human chromatin. Proc. Natl. Acad. Sci. USA.

[41] Izumikawa K., Yanagida M., Hayano T., Tachikawa H., Komatsu W., Shimamoto A., Futami K., Furuichi Y., Shinkawa T., Yamauchi Y. (2008). Association of human DNA helicase RecQ5beta with RNA polymerase II and its possible role in transcription. Biochem. J..

[42] Zhou G., Liu Y., Wu S.Y., Tie F., Lou H., Chiang C.M., Luo G. (2010). Purification of a novel RECQL5-SWI/SNF-RNAPII super complex. Int. J. Biochem. Mol. Biol..

[43] Islam M.N., Fox D., Guo R., Enomoto T., Wang W. (2010). RecQL5 Promotes Genome Stabilization through Two Parallel Mechanisms—Interacting with RNA Polymerase II and Acting as a Helicase. Mol. Cell. Biol..

[44] Li M., Xu X., Liu Y. (2011). The SET2-RPB1 interaction domain of human RECQ5 is important for transcription-associated genome stability. Mol. Cell. Biol..

[45] Kanagaraj R., Huehn D., MacKellar A., Menigatti M., Zheng L., Urban V., Shevelev I., Greenleaf A.L., Janscak P. (2010). RECQ5 helicase associates with the C-terminal repeat domain of RNA polymerase II during productive elongation phase of transcription. Nucleic Acids Res..

[46] Aygün O., Xu X., Liu Y., Takahashi H., Kong S.E., Conaway R.C., Conaway J.W., Svejstrup J.Q. (2009). Direct inhibition of RNA polymerase II transcription by RECQL5. J. Biol. Chem..

[47] Kassube S.A., Jinek M., Fang J., Tsutakawa S., Nogales E. (2013). Structural mimicry in transcription regulation of human RNA polymerase II by the DNA helicase RECQL5. Nat. Struct. Mol. Biol..

[48] Phatnani H.P., Greenleaf A.L. (2006). Phosphorylation and functions of the RNA polymerase II CTD. Genes Dev..

[49] Saponaro M., Kantidakis T., Mitter R., Kelly G.P., Heron M., Williams H., Söding J., Stewart A., Svejstrup J.Q. (2014). RECQL5 controls transcript elongation and suppresses genome instability associated with transcription stress. Cell.

[50] Aguilera A., Garcia-Muse T. (2012). R loops: From transcription byproducts to threats to genome stability. Mol. Cell.

[51] Wang J.C. (2002). Cellular roles of DNA topoisomerases: A molecular perspective. Nat. Rev. Mol. Cell Biol..

[52] Imai K., Yamamoto H. (2008). Carcinogenesis and microsatellite instability: The interrelationship between genetics and epigenetics. Carcinogenesis.

[53] Takahashi T., Burguiere-Slezak G., Van der Kemp P.A., Boiteux S. (2011). Topoisomerase 1 provokes the formation of short deletions in repeated sequences upon high transcription in Saccharomyces cerevisiae. Proc. Natl. Acad. Sci. USA.

[54] Kim N., Huang S.N., Williams J.S., Li Y.C., Clark A.B., Cho J.E., Kunkel T.A., Pommier Y., Jinks-Robertson S. (2011). Mutagenic processing of ribonucleotides in DNA by yeast topoisomerase I. Science.

[55] Li M., Pokharel S., Wang J.T., Xu X., Liu Y. (2015). RECQ5-dependent SUMOylation of DNA topoisomerase I prevents transcription-associated genome instability. Nat. Commun..

[56] Tuduri S., Crabbe L., Conti C., Tourriere H., Holtgreve-Grez H., Jauch A., Pantesco V., De Vos J., Thomas A., Theillet C. (2009). Topoisomerase I suppresses genomic instability by preventing interference between replication and transcription. Nat. Cell Biol..

[57] Helmrich A., Ballarino M., Nudler E., Tora L. (2013). Transcription-replication encounters, consequences and genomic instability. Nat. Struct. Mol. Biol..

[58] Yeeles J.T. (2014). Discontinuous leading-strand synthesis: A stop-start story. Biochem. Soc. Trans..

[59] Helmrich A., Ballarino M., Tora L. (2011). Collisions between replication and transcription complexes cause common fragile site instability at the longest human genes. Mol. Cell.

[60] Barlow J.H., Faryabi R.B., Callen E., Wong N., Malhowski A., Chen H.T., Gutierrez-Cruz G., Sun H.W., McKinnon P., Wright G. (2013). Identification of early replicating fragile sites that contribute to genome instability. Cell.

[61] Helmrich A., Stout-Weider K., Hermann K., Schrock E., Heiden T. (2006). Common fragile sites are conserved features of human and mouse chromosomes and relate to large active genes. Genome Res..

[62] Debacker K., Kooy R.F. (2007). Fragile sites and human disease. Hum. Mol. Genet..

[63] Drusco A., Pekarsky Y., Costinean S., Antenucci A., Conti L., Volinia S., Aqeilan R.I., Huebner K., Zanesi N. (2011). Common fragile site tumor suppressor genes and corresponding mouse models of cancer. J. Biomed. Biotechnol..

[64] Hazan I., Hofmann T.G., Aqeilan R.I. (2016). Tumor Suppressor Genes within Common Fragile Sites Are Active Players in the DNA Damage Response. PLoS Genet..

[65] Aygun O., Svejstrup J.Q. (2010). RECQL5 helicase: Connections to DNA recombination and RNA polymerase II transcription. DNA Repair.

[66] Li M., Xu X., Chang C.W., Zheng L., Shen B., Liu Y. (2018). SUMO2 conjugation of PCNA facilitates chromatin remodeling to resolve transcription-replication conflicts. Nat. Commun..

[67] Li M., Xu X., Chang C.W., Liu Y. (2020). TRIM28 functions as the SUMO E3 ligase for PCNA in prevention of transcription induced DNA breaks. Proc. Natl. Acad. Sci. USA.

[68] Urban V., Dobrovolna J., Hühn D., Fryzelkova J., Bartek J., Janscak P. (2016). RECQ5 helicase promotes resolution of conflicts between replication and transcription in human cells. J. Cell Biol..

[69] Uhlén M., Fagerberg L., Hallström B.M., Lindskog C., Oksvold P., Mardinoglu A., Sivertsson Å., Kampf C., Sjöstedt E., Asplund A. (2015). Proteomics. Tissue-based map of the human proteome. Science.

[70] Lao V.V., Welcsh P., Luo Y., Carter K.T., Dzieciatkowski S., Dintzis S., Meza J., Sarvetnick N.E., Monnat R.J., Loeb L.A. (2013). Altered RECQ Helicase Expression in Sporadic Primary Colorectal Cancers. Transl. Oncol..

[71] Lin Y., Chen H., Wang X., Xiang J., Wang H., Peng J. (2020). Mining the role of RECQL5 in gastric cancer and seeking potential regulatory network by bioinformatics analysis. Exp. Mol. Pathol..

[72] Lin Y., Wang H., Wang X., Li M., Chen H., Peng J. (2020). Low expression of RecQ-like helicase 5 is associated with poor prognosis in patients with gastric cancer. Oncol. Lett..

[73] Hu Y., Lu X., Luo G. (2010). Effect of Recql5 deficiency on the intestinal tumor susceptibility of Apc(min) mice. World J. Gastroenterol..

[74] Wu J., Zhi L., Dai X., Cai Q., Ma W. (2015). Decreased RECQL5 correlated with disease progression of osteosarcoma. Biochem. Biophys. Res. Commun..

[75] Aqeilan R.I., Kuroki T., Pekarsky Y., Albagha O., Trapasso F., Baffa R., Huebner K., Edmonds P., Croce C.M. (2004). Loss of WWOX expression in gastric carcinoma. Clin. Cancer Res..

[76] Huebner K., Croce C.M. (2003). Cancer and the FRA3B/FHIT fragile locus: It’s a HIT. Br. J. Cancer.

[77] Fewings E., Larionov A., Redman J., Goldgraben M.A., Scarth J., Richardson S., Brewer C., Davidson R., Ellis I., Evans D.G. (2018). Germline pathogenic variants in PALB2 and other cancer-predisposing genes in families with hereditary diffuse gastric cancer without CDH1 mutation: A whole-exome sequencing study. Lancet Gastroenterol. Hepatol..

[78] Tavera-Tapia A., de la Hoya M., Calvete O., Martin-Gimeno P., Fernández V., Macías J.A., Alonso B., Pombo L., de Diego C., Alonso R. (2019). RECQL5: Another DNA helicase potentially involved in hereditary breast cancer susceptibility. Hum. Mutat..

[79] Marchena-Perea E.M., Salazar-Hidalgo M.E., Gómez-Sanz A., Arranz-Ledo M., Barroso A., Fernández V., Tejera-Pérez H., Pita G., Núñez-Torres R., Pombo L. (2022). A Large Case-Control Study Performed in Spanish Population Suggests That RECQL5 Is the Only RECQ Helicase Involved in Breast Cancer Susceptibility. Cancers.

[80] Arora A., Abdel-Fatah T.M.A., Agarwal D., Doherty R., Croteau D.L., Moseley P.M., Hameed K., Green A., Aleskandarany M.A., Rakha E.A. (2016). Clinicopathological and prognostic significance of RECQL5 helicase expression in breast cancers. Carcinogenesis.

[81] Zhu X., Chen H., Yang Y., Xu C., Zhou J., Zhou J., Chen Y. (2018). Distinct prognosis of mRNA expression of the five RecQ DNA-helicase family members—RECQL, BLM, WRN, RECQL4, and RECQL5—In patients with breast cancer. Cancer Manag. Res..

[82] Ochs-Balcom H.M., Thompson C.L., Plummer S., Luo G., Tucker T.C., Casey G., Li L. (2010). A RecQ Protein-like 5 Haplotype is Associated with Colon Cancer. Gastroenterol. Res..

[83] Zhi L.-Q., Ma W., Zhang H., Zeng S.-X., Chen B. (2014). Association of RECQL5 gene polymorphisms and osteosarcoma in a Chinese Han population. Tumor Biol..

[84] He Y.J., Qiao Z.Y., Gao B., Zhang X.H., Wen Y.Y. (2014). Association between RECQL5 genetic polymorphisms and susceptibility to breast cancer. Tumor Biol..

[85] Stirnweiss A., Oommen J., Kotecha R.S., Kees U.R., Beesley A.H. (2017). Molecular-genetic profiling and high-throughput in vitro drug screening in NUT midline carcinoma-an aggressive and fatal disease. Oncotarget.

[86] Das R., Kundu S., Laskar S., Choudhury Y., Ghosh S.K. (2018). Assessment of DNA repair susceptibility genes identified by whole exome sequencing in head and neck cancer. DNA Repair.

[87] Wang X., Liu Z. (2018). Systematic meta-analysis of genetic variants associated with osteosarcoma susceptibility. Medicine.

[88] Qi Y., Zhou X. (2014). Haplotype analysis of RECQL5 gene and laryngeal cancer. Tumor Biol..

[89] Yuan Y., Yao S., Luo G.H., Zhang X.Y. (2021). Impact of metabolism-related mutations on the heart rate of gastric cancer patients after peritoneal lavage. World J. Clin. Cases.

[90] Dong Y., Huang Y., Lu T. (2015). Single nucleotide polymorphism in the RECQL5 gene increased osteosarcoma susceptibility in a Chinese Han population. Genet. Mol. Res..

[91] Yuan D., Tian J., Fang X., Xiong Y., Banskota N., Kuang F., Zhang W., Duan H. (2022). Epidemiological Evidence for Associations Between Genetic Variants and Osteosarcoma Susceptibility: A Meta-Analysis. Front. Oncol..

[92] Tunyasuvunakool K., Adler J., Wu Z., Green T., Zielinski M., Žídek A., Bridgland A., Cowie A., Meyer C., Laydon A. (2021). Highly accurate protein structure prediction for the human proteome. Nature.

[93] Varadi M., Anyango S., Deshpande M., Nair S., Natassia C., Yordanova G., Yuan D., Stroe O., Wood G., Laydon A. (2022). AlphaFold Protein Structure Database: Massively expanding the structural coverage of protein-sequence space with high-accuracy models. Nucleic Acids Res..

[94] Ochoa D., Jarnuczak A.F., Viéitez C., Gehre M., Soucheray M., Mateus A., Kleefeldt A.A., Hill A., Garcia-Alonso L., Stein F. (2020). The functional landscape of the human phosphoproteome. Nat. Biotechnol..

[95] Tang Y., Wang Q., Zhang W.K., Liu Y.X., Zheng Z.F., Fan L.L., Liu L., He J. (2023). Case report: A novel mutation of RecQ-like helicase 5 in a Chinese family with early myocardial infarction, coronary artery disease, and stroke hemiplegia. Front. Genet..

[96] Choi Y.Y., Shin S.J., Lee J.E., Madlensky L., Lee S.T., Park J.S., Jo J.H., Kim H., Nachmanson D., Xu X. (2021). Prevalence of cancer susceptibility variants in patients with multiple Lynch syndrome related cancers. Sci. Rep..

[97] Xie X., Zheng Y.Y., Adi D., Yang Y.N., Ma Y.T., Li X.M., Fu Z.Y., Ma X., Liu F., Yu Z.X. (2016). Exome Sequencing in a Family Identifies RECQL5 Mutation Resulting in Early Myocardial Infarction. Medicine.

[98] Etxebarria A., Palacios L., Stef M., Tejedor D., Uribe K.B., Oleaga A., Irigoyen L., Torres B., Ostolaza H., Martin C. (2012). Functional characterization of splicing and ligand-binding domain variants in the LDL receptor. Hum. Mutat..

[99] Ye H., Zhao Q., Huang Y., Wang L., Liu H., Wang C., Dai D., Xu L., Ye M., Duan S. (2014). Meta-analysis of low density lipoprotein receptor (LDLR) rs2228671 polymorphism and coronary heart disease. Biomed. Res. Int..

[100] Sorriento D., Iaccarino G. (2019). Inflammation and Cardiovascular Diseases: The Most Recent Findings. Int. J. Mol. Sci..

[101] Liao W.Q., Qi Y.L., Wang L., Dong X.M., Xu T., Ding C.D., Liu R., Liang W.C., Lu L.T., Li H. (2015). Recql5 protects against lipopolysaccharide/D-galactosamine-induced liver injury in mice. World J. Gastroenterol..

[102] https://www.ncbi.nlm.nih.gov/clinvar/?term=RECQL5%5Bgene%5D&redir=gene.

[103] Fu W., Ligabue A., Rogers K.J., Akey J.M., Monnat R.J. (2017). Human RECQ Helicase Pathogenic Variants, Population Variation and “Missing” Diseases. Hum. Mutat..

[104] Hu Y., Lu X., Zhou G., Barnes E.L., Luo G. (2009). Recql5 plays an important role in DNA replication and cell survival after camptothecin treatment. Mol. Biol. Cell.

[105] Chen E., Ahn J.S., Sykes D.B., Breyfogle L.J., Godfrey A.L., Nangalia J., Ko A., DeAngelo D.J., Green A.R., Mullally A. (2015). RECQL5 Suppresses Oncogenic JAK2-Induced Replication Stress and Genomic Instability. Cell Rep..

[106] Patterson K., Arya L., Bottomley S., Morgan S., Cox A., Catto J., Bryant H.E. (2016). Altered RECQL5 expression in urothelial bladder carcinoma increases cellular proliferation and makes RECQL5 helicase activity a novel target for chemotherapy. Oncotarget.

[107] Chakraborty S., Dutta K., Gupta P., Das A., Das A., Ghosh S.K., Patro B.S. (2021). Targeting RECQL5 Functions, by a Small Molecule, Selectively Kills Breast Cancer in Vitro and in Vivo. J. Med. Chem..

